# Gendered violences and rule of/by law in Cambodia

**DOI:** 10.1177/2043820616655017

**Published:** 2016-07-22

**Authors:** Katherine Brickell

**Affiliations:** University of London, UK

**Keywords:** Cambodia, domestic violence, forced eviction, governance, law, legal geographies

## Abstract

This intervention is based on research in Cambodia on domestic violence and forced eviction. It draws on the distinction between rule of/by law to examine women’s experiences of rights claiming. While ‘rule *of* law’ is a value to be respected and a mechanism via which to guarantee justice and human rights to all citizens, ‘rule *by* law’ is a distortion that is more easily conceived of as an instrument of power and oppression. The intervention’s emphasis on the blurring between these distinctions highlights the stark absence of feminist geography work in the growing field of legal geographies.

Gendered violences take myriad forms, often coexisting within the lives of single individuals, families and communities. In this response, I explore two such manifestations that speak to corporeal, material and symbolic dimensions of violence perpetrated primarily against women. Both forced eviction and domestic violence are injustices which sit at the forefront of current international concern, the former described by UN-Habitat (2011) as a ‘global crisis’, the latter priority theme of the 2013 UN Convention on the Status of Women and subject of a UK Select Committee to which I contributed (Brickell, 2013). Both forced eviction and domestic violence are also ambiguously condoned/sanctioned, prohibited/enacted, through law.

The European Commission for Democracy Through Law (2011) distinguishes between two modes of law. ‘Rule *of* law’ is a value to be respected and a mechanism via which to guarantee justice and human rights to all citizens. ‘Rule *by* law’, meanwhile, is a distortion that is more easily conceived of as an instrument of power and oppression. While nearly a decade earlier, Holmes (2003: 49) noted that ‘rule of law and rule by law occupy a single continuum and do not present mutually exclusive options’, he also recognized that this ‘does not require us to abandon the important distinction’. In Cambodia, the main country focus of my research, the line between rule *of* law and rule *by* law potently testifies to the porosity of home and gendered violences that are experienced on an everyday basis within and beyond its limits. As Delaney (2015: 99) writes in the first *Progress* report on legal geography to be published, ‘“Law” draws lines, constructs insides and outsides, assigns legal meanings to lines, and attaches legal consequences to crossing them’. Despite the stark absence of feminist geography work in the growing field of legal geographies, my commentary here highlights the pertinence of such evocations in relation to gendered violences.

## Forced eviction

The bulldozed line of a wall that once sheltered a family in Boeung Kak speaks to the stark absence of rule *of* law and the prevalence, by contrast, of rule *by* law. The community is the most high-profile case of forced eviction in Cambodia after the lake and surrounding area was illegally leased to a Chinese-backed private development company owned by a Cambodian Senator and then flooded. Springer (2013: 608) has argued that forced eviction in Cambodia is ‘an imposition that serves to legitimize violences of property’. This I contend is a *gendered* violence of property. While it is undeniable that forced eviction has an impact on both men and women’s lives, my research suggests that it is women who are disproportionately affected. Based on testimonies from around the world, COHRE (2010) document how forced eviction generates a host of vulnerabilities for women, including, but not limited to discrimination in respect to property rights and homeownership; exclusion from decision-making and consultation processes due to cultural barriers or gender-specific roles and isolation from social networks of support.

Physical, psychological and/or economic violence from evictors and/or family members is also a common occurrence within the context of forced eviction (COHRE, 2010). In Boeung Kak, this is particularly the case for women who publically contest their dispossession. Abuse by armed police, private forces and company employees have been systematic. Multiple pregnancy miscarriages from authority barbarism have sadly become a recurrent feature. Yet while these acts are in stark contravention of human rights law, the women have themselves been actively pursued via rule *by* law in courts under the grip of state power. The government essentially monopolizes the discursive power of law and silences those who too brightly shine light on state complicity in violence against women. Thirteen Boeung Kak women were jailed in May 2012, the day after ‘illegally occupying’ land, as they tried to rebuild. Later, a fellow group member, Yorm Bopha, was spuriously convicted of intentional violence with aggravating circumstances and sentenced to 3 years in prison in December 2012. Going on to be named prisoner of conscience by Amnesty International, pressure saw her release on bail in November 2013. Since this time, however, the women have been subject to further arrest and imprisonment. Through the means of traffic law, seven received 1-year jail sentences just 24 hours after their arrest for allegedly blocking a main road outside Phnom Penh’s City Hall in November 2014. Embracing ‘active citizenship’, claiming legally and morally enforceable rights in relation to the state, is a dangerous affair in Cambodia. As Jennifer Hyndman (2001: 214) qualifies, ‘state security and human security are not necessarily synonymous’. As I have detailed elsewhere too (Brickell, 2014a: np), ‘Women’s public actions [also] reverberate back into the home, profoundly influencing conjugal and parental relationships’. The interviews with Boeung Kak women and their husbands strongly indicate that marital strain and/or breakdown has become a further source of disruption in peoples’ lives. Their very pursuit of intimate security via active citizenship has, in the short-term at least, sacrificed it ironically further.

## Domestic violence

At the same time that the Cambodian government has wielded rule *by* law *against* women, they have engaged in legal reform to prevent and redress domestic violence. Somewhat conversely then, while the government have brought *dis*harmony to Cambodian homes – and particularly to women – through forced eviction, they have also looked to engender domestic harmony, a goal enshrined in legal rhetoric. Article 1 in the 2005 Law on the Prevention of Domestic Violence and the Protection of Victims includes a clause, which defines its purpose to ‘preserve the harmony within the households in line with the Nation’s good custom and tradition …’. The 2005 Law forms the basis of a study (2012–2015) funded by the ESRC and UK Department for International Development that I led. It has explored the hiatus between legal reform and transformative change for women. The mixed-method findings from two provinces, including a large-scale household survey, participatory video drama workshops and in-depth interviews consistently brings to the fore the normative social pressure exerted on women to uphold the cultural logic of harmony and to ‘endure’ for the sake of family unity rather than end the cycle of violence through law.

‘Governing intimacy’ to use Oswin and Olund’s (2010: 62) turn of phrase is evident in my ongoing research. While a positive move towards rule *of* law and justice for victims, it is important not to ignore the lingering danger of rule *by* law within legal discourse. Women who experience extreme physical violence are repeatedly told to ‘reconcile’ with their spouses by community authorities from whom they seek legal assistance. The local term for reconciliation *samroh samruol* has the meaning to smooth over and seek harmony 34–23‘‘12111111. This is ordinarily sought through a meeting orchestrated by a village leader who tries to encourage compromise between parties to reach an agreement marked verbally or by a promissory note (*liket sanya*). As the official glossary to domestic violence Law reads, this meeting in the case of ‘minor’ domestic violence is designed to allow and facilitate the ‘communication process between quarrelling parties that aims at maintaining family life’ (Royal Government of Cambodia (RGC), 2007: 11). As I elaborate (Brickell, 2015), governmental involvement in lawmaking and their championing of reconciliation has arguably aggravated the potential for further physical violence. It also heightened symbolic violence through the de facto hushing of women. As Bourdieu (1987: 812) outlines, ‘Symbolic violence implies the imposition of such principles of division, and more generally of any symbolic representations (languages, conceptualizations, portrayals), on recipients who have little choice about whether to accept or reject them’. From one perspective then, domestic violence law diminishes the ability of women to claim their rights and redress.

## Concluding thoughts

In this short piece, I have highlighted a range startling fault lines. First, while the 2005 domestic violence law shows a political willingness, in relative terms at least, to tackle gendered insecurity through rule *of* law, the Cambodian government have concurrently used rule *by* law against women who have sought to address gendered insecurities of forced eviction brought to them by the very same government. Second, while legal rhetoric on domestic violence links the security of women to the defence of custom and tradition through rule *of* law, on the ground this translates into an onus on women to remain within abusive relationships. This creates with it a further hiatus between what is permissible under law and what is possible in practice. Together forced eviction, and domestic violence, highlight the divergence between the rhetoric of human rights and the reality of gendered injustice suffered. They powerfully indicate how women’s lives and the domestic are influenced, and they influence a wider politic. And both speak to feminist engagements with law (largely beyond Geography) which ‘reveal the complex relationship between legal framings and process, and social domains, practices, and institutions’ so much so that the legal sphere can be simultaneously defined as ‘a site of oppression and an important means of social transformation’ (Cornwall, 2013: ix).

Geographers have a valuable role to play in pushing forward research where the fields of legal geographies, feminist geopolitics and geographies of home have the potency to further reveal the precarities of everyday life and their gendered dimensions.
